# A combinational approach of multilocus sequence typing and other molecular typing methods in unravelling the epidemiology of *Erysipelothrix rhusiopathiae* strains from poultry and mammals

**DOI:** 10.1186/s13567-015-0216-x

**Published:** 2015-07-21

**Authors:** Traute Janßen, Matthias Voss, Michael Kühl, Torsten Semmler, Hans-Christian Philipp, Christa Ewers

**Affiliations:** Center for Infection Medicine, Institute of Microbiology and Epizootics, Freie Universität Berlin, Robert-von-Ostertag-Straße 7-13, 14163 Berlin, Germany; RIPAC-LABOR GmbH, Am Mühlenberg 11, 14476 Potsdam, Germany; Lohmann Tierzucht GmbH, Cuxhaven, Germany; Institute of Animal Hygiene and Environmental Health, Freie Universität Berlin, Robert-von-Ostertag-Straße 7-13, 14163 Berlin, Germany; Robert Koch Institute, Nordufer 20, 13353 Berlin, Germany; Boehringer Ingelheim Veterinary Research Center GmbH & Co. KG, Bemeroder Straße 31, 30559 Hannover, Germany; Institute of Hygiene and Infectious Diseases of Animals, Justus-Liebig-Universität Giessen, Frankfurter Str. 85-89, 35392 Giessen, Germany

## Abstract

*Erysipelothrix rhusiopathiae* infections re-emerged as a matter of great concern particularly in the poultry industry. In contrast to porcine isolates, molecular epidemiological traits of avian *E. rhusiopathiae* isolates are less well known. Thus, we aimed to (i) develop a multilocus sequence typing (MLST) scheme for *E. rhusiopathiae*, (ii) study the congruence of strain grouping based on pulsed-field gel electrophoresis (PFGE) and MLST, (iii) determine the diversity of the dominant immunogenic protein SpaA, and (iv) examine the distribution of genes putatively linked with virulence among field isolates from poultry (120), swine (24) and other hosts (21), including humans (3). Using seven housekeeping genes for MLST analysis we determined 72 sequence types (STs) among 165 isolates. This indicated an overall high diversity, though 34.5% of all isolates belonged to a single predominant ST-complex, STC9, which grouped strains from birds and mammals, including humans, together. PFGE revealed 58 different clusters and congruence with the sequence-based MLST-method was not common. Based on polymorphisms in the N-terminal hyper-variable region of SpaA the isolates were classified into five groups, which followed the phylogenetic background of the strains. More than 90% of the isolates harboured all 16 putative virulence genes tested and only *intI*, encoding an internalin-like protein, showed infrequent distribution. MLST data determined *E. rhusiopathiae* as weakly clonal species with limited host specificity. A common evolutionary origin of isolates as well as shared SpaA variants and virulence genotypes obtained from avian and mammalian hosts indicates common reservoirs, pathogenic pathways and immunogenic properties of the pathogen.

**Electronic supplementary material:**

The online version of this article (doi:10.1186/s13567-015-0216-x) contains supplementary material, which is available to authorized users.

## Introduction

*Erysipelothrix rhusiopathiae* is a ubiquitous gram-positive bacterial organism which causes erysipelas in mammals and birds, especially in pigs and poultry. Healthy pigs carrying *E. rhusiopathiae* in their lymphoid tissues have been suggested as a reservoir of the pathogen [1]. The genus *Erysipelothrix* contains two main species: *E. rhusiopathiae* (including serotypes 1a, 1b, 2, 4, 5, 6, 8, 9, 11, 12, 15, 16, 17, 19, 21 and N) and *E. tonsillarum* (serotypes 3, 7, 10, 14, 20, 22 and 23), the latter being isolated from apparently healthy swine and considered to be non-pathogenic [1-4].

*E. rhusiopathiae* was first recognized as a human pathogen causing localized cutaneous lesions, called erysipeloid, and sporadic cases of generalized cutaneous forms or septicaemia, often associated with endocarditis [2,5]. Erysipelas in swine occurs in different forms characterized by septicaemia often resulting in sudden death (acute form), cutaneous lesions (sub-acute form), polyarthritis and endocarditis (chronic form) [1,2,6]. In recent years, Erysipelas in poultry re-emerged and here, turkeys are most seriously affected and suffer from cyanotic skin to haemorrhages and petechiae in the breast and leg muscles. Following the change from conventional (battery) cage system to alternative housing systems, problems due to *E. rhusiopathiae* also increased in laying hen [2,6-12].

While attenuated live vaccines and bacterins are commercially available to protect pigs and sheep, in Germany there is no licensed vaccine to prevent erysipelas in turkeys and laying hen and this is why the application of autogenous vaccines and/or antimicrobial therapy is the ultimate way to combat the disease [1,2,9]. One of the most promising vaccine candidates is SpaA, a surface protein with high immunogenic properties [13-16]. Based on amino acid sequence similarities the Spa proteins can be classified into 3 molecular variants, termed SpaA, SpaB, and SpaC and the occurrence of these variants has been found to be basically associated with the serotype of a strain [14]. Particularly the N-terminal half of the hyper-variable region of the Spa protein is important for specific immunity, and strains with different *spaA* gene variants obviously differ in pathogenicity [17].

*E. rhusiopathiae* strains vary considerably in virulence but only little is known about pathogenic mechanisms and the presence and distribution of virulence factors [2]. Different factors, including neuraminidase, capsule, hemolysin, hyaluronidase, adhesins and the cell wall lipoprotein EwlA have been suggested to be involved in the pathogenesis of the disease [2]. The availability of the genome sequence of *E. rhusiopathiae* strain Fujisawa (Acc.-No. AP012027.1) offers the opportunity for the *in silico*-identification of additional genes that have been linked with virulence in other bacterial species before. The coding sequence ERH_1472 was for example annotated as internalin gene *intI*, which in case of *Listeria monocytogenes* contributes to the invasion of the bacteria into epithelial cells [18].

Various studies have been performed to determine the clonal relatedness of *E. rhusiopathiae* strains [19-25]. Data from multilocus enzyme electrophoresis (MEE) [20], restriction fragment length polymorphism (RFLP) analyses [19], amplified fragment length polymorphism (AFLP) [21], randomly amplified polymorphic DNA methods (RAPD) [23,25], and pulsed-field gel electrophoresis (PFGE) [22,24] suggested that *E. rhusiopathiae* strains might be genetically diverse even among isolates belonging to the same serotype. Although the relevance of Erysipelas in poultry increased over the last years, intense epidemiological studies on *E. rhusiopathiae* from poultry combining established typing methods such as PFGE, Spa typing and virulence gene typing are limited [10,22] Moreover, there is currently no established scheme to study this bacterial species by Multilocus sequence typing (MLST), a widely applied method to determine the phylogeny of a bacterial population based on sequence analysis of representative genes of the bacterial core genome [26].

The overall objective of this study was to provide a deeper insight into the population structure of *E. rhusiopathiae* by developing an MLST scheme and determining sequence types of 165 *E. rhusiopathiae* strains predominantly isolated from poultry and also from mammalian hosts, including few human strains. The congruence of multilocus sequence types of individual strains with their grouping into PFGE clusters and their surface protective antigen (Spa) protein variant was evaluated and the distribution of 16 genes putatively linked with the pathogenic properties of the strains was investigated.

## Materials and methods

### Bacterial strains and species designation

A total of 165 *E. rhusiopathiae* strains primarily from Germany (136) and from a few other countries (29) were included. The major focus was on isolates from avian sources (chicken, 71; turkey, 43; other birds, 6), while isolates from pigs (*n* = 36) and other animal species (*n* = 6) and humans (3) were included as well. Bacterial strains were isolated from organ and blood samples from diseased animals and from blood samples in case of the human strains. In case of outbreak situations, only one strain per flock was sampled. Strains obtained from recurrent disease events in identical flocks were only included if six months had passed since the previous event or if they revealed different banding patterns, as initially determined by pulsed-field gel electrophoresis (data not shown).

*E. rhusiopathiae* strains and the non-pathogenic species *E. tonsillarum* were differentiated by a species-specific PCR [27]. Reference strains *E. rhusiopathiae* ATCC 19414^T^, *E. tonsillarum* DSM 14972^T^ (ATCC 43339^T^), and *E. inopinata* DSM 15511^T^ as well as two field strains of *E. tonsillarum* were also included. The reference strains were purchased from the Leibniz Institute DSMZ-German Collection of Microorganisms and Cell Cultures (Braunschweig, Germany). All strains were stored at −80 °C in liquid medium (brain heart infusion broth) supplemented with 20% glycerol until used.

### DNA purification

The total DNA of *E. rhusiopathiae* was purified using a protocol described previously [28]. Instead of using a 1.5 mL bacterial culture grown in BHI for 24 h, as recommended in the protocol, we took 20 mL of an overnight culture to obtain a higher amount of DNA.

DNA concentration was determined by UV/VIS spectrophotometry (NanoDrop®ND1000 Spectrophotometer, Thermo Fisher Scientific). Aliquots (20 ng/μL) were stored at −20 °C until further use.

### Genes for MLST and nucleotide sequencing of gene fragments

To establish an MLST scheme, genes were selected based on published protein sequences of *E. rhusiopathiae,* located in the published contigs of the reference strain *E. rhusiopathiae* ATCC 19414^T^ (project ID: 38421), sequenced by the National Institute of Animal Health, Japan, in cooperation with the Dragon Genomics Center, Takara Bio Inc., Japan. Based on reversed translated protein sequences, primers were designed to amplify gene fragments of housekeeping genes *gpsA* (glycerol-3-phophate-dehydrogenase), *recA* (recombinase A), *purA* (adenylosuccinate synthetase), *pta* (phosphate acetyl-transferase), *prsA* (ribose-phosphate-pyrophosphokinase), *galK* (galactokinase), and *ldhA* (D-lactate dehydrogenase) (Table 1). Genes were chosen in line with MLST schemes for other bacteria taking into account (i) a gene size > 700 bp, (ii) a regular distribution of genes among the *E. rhusiopathiae* genome, and (iii) that predicted proteins should be under stabilizing selection.

**Table 1:** Primer sets used to amplify and sequence *E. rhusiopathiae* housekeeping genes and genetic diversity of gene loci used for MLST analysis

Gene	Primer sequence [5′-3′]	Location within gene^a^	Amplicon size PCR (bp)	Allele size MLST (bp)	Mean GC content	No. of alleles	No. of variable sites (%)	*d*_*N*_ */d*_*S*ratio_	Simpsons index of diversity (*D*)
*gpsA*	fw: AGTTATGATGTGGGGACG	75-745	671	540	38.3%	9	11 (2.0)	0.3610	0.608
	rv: TAGCTGTAACGACGAGATCG								
*recA*	fw: TTCGGTAGAATAATCTCGCG	38-913	876	640	39.7%	9	13 (1.6)	0.0207	0.775
	rv: TGCTATTAGTTCAGGGTCG								
*purA*	fw: GATGTTTATGAGGAAGCGC	331-1066	736	611	39.1%	14	11 (1.8)	0.5690	0.764
	rv: AACGCATTGATTGTTGCCC								
*pta*	fw: TGCTGCAGTACGTTTAGC	90-793	704	544	36.6%	9	12 (2.2)	0.0419	0.579
	rv: AGACACGTGCATTACCTG								
*prsA*	fw: ACAAGTTCACCAGTAAGTG	190-952	763	575	39.3%	6	4 (0.7)	0.0654	0.657
	rv: AGAGTGTACTTACAGGAGT								
*galK*	fw: TATTCCTAATGGAGCGGG	357-1041	685	554	36.8%	13	10 (1.8)	0.0766	0.740
	rv: AATCGCAATCGCACATCC								
*ldhA*	fw: AACGGATATGAAGCTGTTGCC	136-783	648	531	40.3%	11	14 (2.6)	0.3390	0.681
	rv: AAGAACATCCAGTCCAACAGC								

^a^As referred to *E. rhusiopathiae* ATCC 19414^T^ (Acc.-No. NZ_ACLK02000001 - NZ_ACLK02000004).

PCRs were performed in a volume of 60 μL containing 60 ng DNA template, 10 pmol of each primer (Sigma Alderich, Munich, Germany), 10 mM dNTP mixture, 10 × PCR buffer including 20 mM MgCl_2_, and 1 U Taq polymerase (all Rapidozym, Berlin, Germany). The PCR conditions were initial denaturation at 95 °C for 3 min followed by 30 cycles of denaturation at 95 °C for 30 s, annealing at 55 °C for 30 s (except for *galK*, for which an annealing temperature of 54 °C was used), and extension at 72 °C for 1 min, with a final extension at 72 °C for 10 min. Amplicons were sequenced by LGC genomics (Berlin, Germany). Sequence analysis was performed with Ridom SeqSphere 1.0.1 [29].

Each unique sequence of a gene fragment was assigned an allele number. These allele numbers were consecutively numbered and combined in order to establish an allelic profile, which defined the sequence type (ST). Using the eBURST program [30] the MLST data set was divided into groups of related STs (clonal complexes resp. ST complexes). We used the most stringent definition of ST complexes, which were defined as groups when six out of seven alleles from the nearest neighbour were the same [31].

The program START version 2 [32], was used to determine the number of variable nucleotide sites and their effects on the amino acid sequence changes. This was done by calculating the ratio of non-synonymous substitutions (*d*_*N*_) to the number of synonymous substitutions (*d*_*S*_) (*d*_*N*_/*d*_*S*_ ratio) [33]. The *d*_*N*_*/d*_*S*_ ratio quantifies the evolutionary pressure on proteins [34] and thus indicates the presence or absence of a selective force on the locus [35].

Simpson’s index of Diversity (*D*) was calculated on the basis of the molecular pattern of the seven loci [36-38]. A Simpson’s index of diversity with 95% confidence intervals (CI_95%_) was calculated for a set of 165 strains. A value close to 1 indicates high diversity, and a value close to 0 reflects little diversity.

### Pulsed-field gel electrophoresis

Pulsed-field gel electrophoresis (PFGE) followed a previously published protocol [24] and was performed to group *E. rhusiopathiae* isolates according to their macrorestriction profiles. PFGE was also applied to discuss the discriminatory power and interpretative value of this band-based method compared with the sequence-based MLST analysis. The chromosomal DNA was digested with SmaI (Fermentas Life Science, Germany). Electrophoresis was carried out in a contour-clamped homogeneous electric field (CHEF- DRIII; Biorad, Munich, Germany). MidRange PFG Marker I (BioLabs, #N3551S) was used as DNA size standard. Band patterns were analysed with the software BioNumerics (Version 6.6, Applied Maths, Belgium). Dendrograms were created by an unweighted pair group matching by an arithmetic averages algorithm (UPGMA) and the Dice coefficient. Optimization was set at 0.9%, band position tolerance at 2.0% [39]. To compare the discriminatory power of MLST and PFGE the Simpson’s index of Diversity (*D*) was used [36-38]. An index of diversity close to 1 indicated a high diversity of the strain collection, whereas a value close to 0 suggested little diversity. We calculated the Simpson’s index of diversity (*D*) with 95% confidence intervals (CI_95%_).

### Detection of virulence-associated genes by Multiplex PCR

Two multiplex PCR protocols were established to screen for genes putatively linked with the virulence of *E. rhusiopathiae*. The selection of the genes was based on (i) previous studies, linking the gene products with a certain pathogenic mechanism [2,40-44] and (ii) publicly available annotation data of coding sequences of *E. rhusiopathiae* (Acc.-No. AP012027.1), determined by whole genome analysis [45] (see Table 2). The sequences of oligonucleotide primers, either obtained from recent publications [14,46] or designed in the present study are given in Table 2. PCRs were performed in a T300 Thermocycler (Biometra, Göttingen, Germany). A 25 μL aliquot contained 3 μL of each of purified chromosomal DNA (20 ng), 20 pmol of each primer pair (Sigma Alderich, Munich, Germany), 5 mM of the four deoxynucleoside triphosphates (Rapidozym, Berlin, Germany), 10 × PCR buffer including 20 mM MgCl_2_, and 0.4 U Dream Green Taq DNA Polymerase (Thermo Fisher Scientific, St. Leon-Rot, Germany). PCR was performed under the following conditions: an initial denaturation step at 94 °C for 10 min followed by 30 cycles of denaturation at 94 °C for 1 min, annealing at 53 °C for 2 min, extension at 72 °C for 7 min, and finally one cycle of extension at 72 °C for 10 min. PCR products were separated by gel electrophoresis in a 1.5% agarose gel containing ethidiumbromid for 90 min at 120 V and analysed by gel documentation system (Easy doc, Herolab, Wiesloch, Germany).

**Table 2:** Oligonucleotide primers used for multiplex PCRs to detect 16 genes putatively linked with virulence of *E. rhusiopathiae*

Gene / locus tag	Gene product / predicted function [reference]	Primer sequence (5′ – 3′) (forward/reverse)^a^	GenBank Acc. No	Localization within gene	Size (bp)	Gene pre-valence (%)
ERH_1356	ABC transporter metal-binding protein / adhesion of host cells [45]	CATGAAGGGTAACACCTTGG/ GGGCGATAAAGTTGCGGTAGAA	NC_015601.1	579 – 787	209	100
*intI-like*	Internalin / invasion of epithelial cells [18,45]	ACAGTTTCGGATACTTCCGG/ ACCCTCGTCATATTTACCAGC	AP012027.1	386 – 714	329	85.5
*rspB*	Rhusiopathiae surface protein /biofilm formation [41]	ATCTTTACCCAATTCGACGT/ ATGAACCCAGTCCAAGATTGG	AB052682.1	6882 – 7287	406	100
*rspA*	Rhusiopathiae surface protein /biofilm formation [41]	ATCGACTGGTATTCAGTTGG/ ATCACGAGACATACCGCCAA	AB052682.1	597 – 1131	535	100
*cap locus*	Capsule / resistance to phagocytosis, intracellular survival [40]	TATCTTTGTAGCGTAGTTGG/ CAATAAAAGGAAATACCAGTGC	D64177	1333 – 1987	645	100
*algI*	Alginate-O-acetyltransferase / resistance to phagocytosis [45]	AGTTATCTTGGACTTGGTCC/ AGATAAGTGCGCATTGATCC	AP012027.1	121 – 887	767	97.6
*ewlA*	Lipoprotein / adhesion to host cells [45]	TAATATTAGATAGCGAGGAAT/ AAGAAAAGGGAGTGTGAATAT	U52850.1	226 –1185	960	100
*nanH*	Neuraminidase / spreading factor, nutritive [44]	ATGAAGCGCTTACATTTGAAT/ TACATAAGGTTGACCAAAGTC	AB019122	295 –1401	1.107	100
*sub*	S8-subtilisin / peptidase [45]	AAGCCTGAGATATCTGCACC/ TTGTACAATTGGATGAGCCG	AP012027.1	1360 –1585	226	100
*sodA*	Superoxide dismutase / antioxidans [45,70]	AGAAGACATCCGCACAGCAGT/ GCATGTTCCCAAACATCAAGA	AP012027.1	195 – 509	315	100
*mviN1*	Integral membrane protein / cell adhesion, transport protein [45]	AAATCATGCTTGTAATGGCGG/ ATTCGACGTTAAAACAACCGC	AP012027.1	565 –1032	378	100
*hep*	Heparinase / inactivation of heparin [45]	ATGGAAGTACCGATCTCACT/ TCATTGTAGCAACATGGCTTC	AP012027.1	997 –1480	484	100
*hlyIII*	Hemolysin / lytic activity on red blood cells [42]	TACGATTGCGACAAAGTGTGCG/ ATGGAAACATAGGGAAGGCTG	AP012027.1	16 – 559	544	100
*fbpA*	Fibronectin-binding protein / adhesion [41]	ATCTCGCCGCTTTTAGAACG/ GCGTCTTCAACTGTTGCTTG	AP012027.1	565 –1166	602	100
*hlyA*	Hyaluronidase / spreading factor [2]	AGGATCACTTACCGCTATGG/ CAGCACTCAGCATGTTCTC	AP012027.1	598 –1538	941	100
*dnaB*	Membrane-, attachment protein / proliferation, adhesion [45]	AATAGCCCCTGATCAAATGG/ CTCTCCTTTACTTAACATCCC	ACLK02000002.1	39 –1155	1.117	99.4

Primers were designed in this study except for the primers to amplify the *cap* locus [46].

^a^In case Ogawa et al. [45] is given as a reference, the predicted function of the gene product has been deduced from genome annotation data of strain Fujisawa. Here, in vitro or in vivo studies would be mandatory to verify the linkage of the genes with the pathogenesis of Erysipelas in different hosts.

**Table 3:** Sequence types (ST) with corresponding allele numbers demonstrated for 165 *E. rhusiopathiae* and number of each ST

ST	*gpsA*	*recA*	*purA*	*pta*	*prsA*	*galK*	*ldhA*	n
1	1	3	1	1	4	4	4	2
2	1	3	1	3	1	1	1	4
3	2	4	3	2	2	2	2	6
4	2	4	2	2	2	5	2	11
5	2	4	4	2	2	2	3	10
6	2	2	2	2	2	2	2	3
7	1	3	1	1	1	1	1	6
8	1	1	1	1	1	4	1	3
9	1	1	1	1	1	1	1	18
10	1	1	6	2	2	2	2	4
11	1	1	1	1	1	1	3	1
12	1	2	1	1	1	4	1	2
13	2	4	2	2	4	6	2	2
14	3	2	1	1	1	3	1	1
15	2	4	7	2	4	6	2	1
16	2	5	8	2	4	2	2	1
17	2	1	6	1	2	5	2	1
18	2	5	5	2	2	2	3	1
19	2	4	9	1	1	1	2	5
20	2	4	4	2	2	2	4	2
21	1	1	1	1	1	7	1	1
22	3	2	1	4	1	3	1	1
23	1	1	1	1	3	4	1	1
24	4	2	6	2	2	2	2	1
25	5	3	2	5	4	1	1	1
26	1	4	1	1	1	5	1	2
27	2	5	3	2	2	2	3	3
28	2	5	6	2	2	2	2	1
29	1	6	1	1	1	4	1	1
30	1	3	11	1	1	1	1	1
31	1	1	1	1	1	1	6	1
32	5	1	1	1	1	1	1	5
33	2	1	3	2	1	1	2	1
34	2	2	6	2	2	5	2	1
35	1	7	12	6	1	5	7	1
36	1	2	6	2	2	5	2	2
37	6	1	1	2	1	4	1	2
38	2	5	10	2	4	5	3	1
39	2	5	3	2	2	5	2	2
40	2	4	1	7	4	2	3	4
41	2	2	2	2	4	2	2	1
42	2	5	3	2	2	8	2	1
ST	*gpsA*	*recA*	*purA*	*pta*	*prsA*	*galK*	*ldhA*	n
43	1	1	1	1	3	1	1	2
44	1	1	1	1	2	1	1	1
45	1	3	1	1	1	1	9	1
46	1	3	13	1	1	1	1	1
47	2	4	2	8	2	6	3	2
48	2	2	2	2	5	1	2	7
49	2	2	14	2	2	2	8	2
50	2	5	2	2	4	5	2	1
51	1	2	2	1	4	10	10	1
52	5	8	1	1	1	1	9	1
53	6	2	1	1	1	9	1	1
54	7	1	1	1	1	1	1	2
55	2	2	3	2	4	2	3	4
56	1	1	1	1	6	4	1	1
57	1	2	1	1	1	2	1	1
58	1	4	1	9	1	5	1	1
59	2	1	3	2	4	4	2	1
60	2	1	3	2	2	5	11	2
61	2	5	1	2	2	11	2	1
62	2	4	9	1	1	12	2	1
63	1	9	1	1	1	4	1	1
64	2	2	6	2	2	2	3	1
65	8	3	2	1	1	1	1	2
66	1	3	13	1	4	1	1	1
67	5	1	2	1	2	1	1	1
68	1	2	6	1	2	2	2	1
69	5	2	6	1	1	4	1	1
70	9	3	3	2	2	13	2	1
71	1	1	1	1	1	2	1	2
72	2	4	3	2	4	2	2	1

### Sequencing of spaA genes

In order to distinguish the three different molecular variants of the Spa protein [14] we performed restriction length polymorphism (RFLP) analysis and single locus sequence typing (SLST) of the *spa* gene. The Spa type variant genes *spaA*, *spaB* and *spaC* were PCR amplified under standard PCR conditions (60 ng template DNA, 10 pmol each primer (Sigma Alderich, Munich, Germany), 10 × PCR buffer including 20 mM MgCl_2_, 5 mM dNTP mixture, and 0.5 U Dream Green Taq DNA Polymerase (Thermo Fisher Scientific, St. Leon-Rot, Germany), using primers Spa-fw (5′- ATGAAAAAGAAAAAACACCTA – 3′), located at position 1–21, and Spa-rv (5′- CTATTTTAAACTTCCATCGTT – 3′), located at position 1881–1862 of serotype 1a reference strain Fujisawa (Acc.-No. AP012027.1). The cycling conditions were 5 min at 94 °C, followed by 30 cycles of 30 s at 94 °C, 30 s at 52 °C and 130 s at 72 °C. Final extension was done at 72 °C for 10 min. Alignment of primers with publicly available *spaB* (Acc.-No. AB238211 - AB238215) and *spaC* (Acc.-No. AB238210) gene sequences of serotype 4, 6, 11, 19, 21, and 18 strains, respectively, revealed that they could amplify all known gene variants correctly. Amplicons were then digested with restriction enzyme *HhaI* overnight at 37 °C. Spa genes revealing different RFLP patterns were double-strand sequenced using the same primers as above and data were analyzed with RidomSeqSphere 1.0.1 [29] and the EditSeq and MegAlign programs of the DNAStar software package (DNASTAR Inc. Madison, Wisconsin, USA). Alignments of nucleotide and amino acid sequences were performed with RidomSeqSphere.

## Results

### Multilocus sequence typing

Allele sizes for the genes included for MLST analysis of 165 *E. rhusiopathiae* strains ranged between 531 bp for *ldhA* to 640 bp for *recA* (Table 1). The mean GC content varied from 36.6% (*pta*) to 40.3% (*ldhA*). Among the 165 investigated isolates with the corresponding 3.995 bp of the concatenated sequences of seven loci, 75 (1.9%) variable sites were confirmed. The number of alleles ranged from six for *prsA* to 14 for *purA*. The nucleotide diversity within the single loci varied between four (0.7%) for *prsA* and 14 (2.6%) for *ldhA,* the diversity index (*D*) for each allele between 0.579 (*pta*) and 0.775 (*recA*). The *d*_*N*_*/d*_*S*_ ratio for all seven loci demonstrated to be < 1 (Table 1).

**Figure 1:**
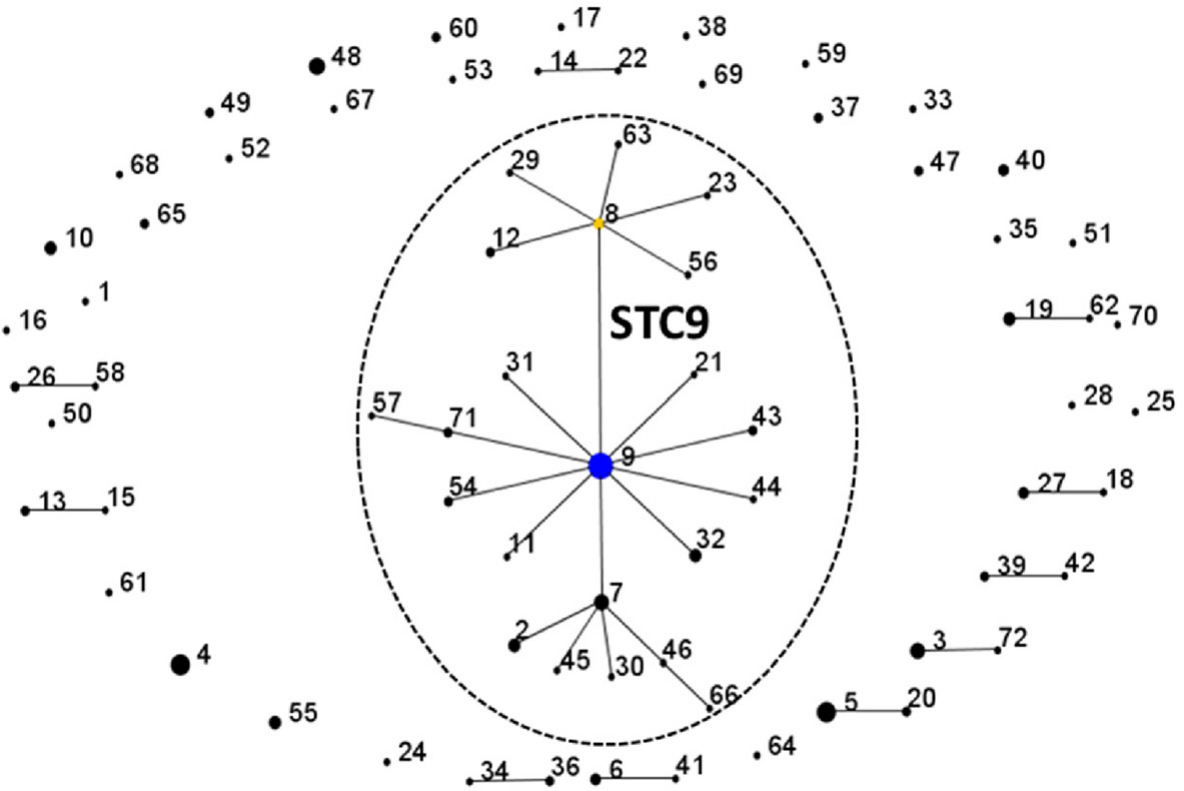
**Population snapshot of 165** ***E. rhusiopathiae*** **isolates assigned to 72 sequence types (STs).** STs connected by a line share six out of seven alleles. The dominant ST complex (STC9) which is named after its predicted founder is highlighted by a dotted circle.

The combination of allele numbers and the corresponding sequence types are shown in Table 3. A list of all strains with their sequence types is available as additional file (see Additional file 1). Of 72 sequence types (STs) assigned to 165 *E. rhusiopathiae* isolates, more than half (58.3%) were singletons. The remaining 29 STs were represented by two or more strains. ST9 was by far the most common sequence type, which grouped 18 isolates (10.9%) from poultry (*n* = 12), pigs (*n* = 3), sheep (*n* = 1), and also from a case of wound infection and a case of endocarditis in humans together. Sequence type 4 (*n* = 11 poultry isolates) and ST5 (*n* = 10 poultry and one pig isolate) were the second most frequent STs. Overall, eBURST analysis based on all allelic profiles revealed a highly divergent population structure among the *E. rhusiopathiae* strains under study (Figure 1).

**Figure 2:**
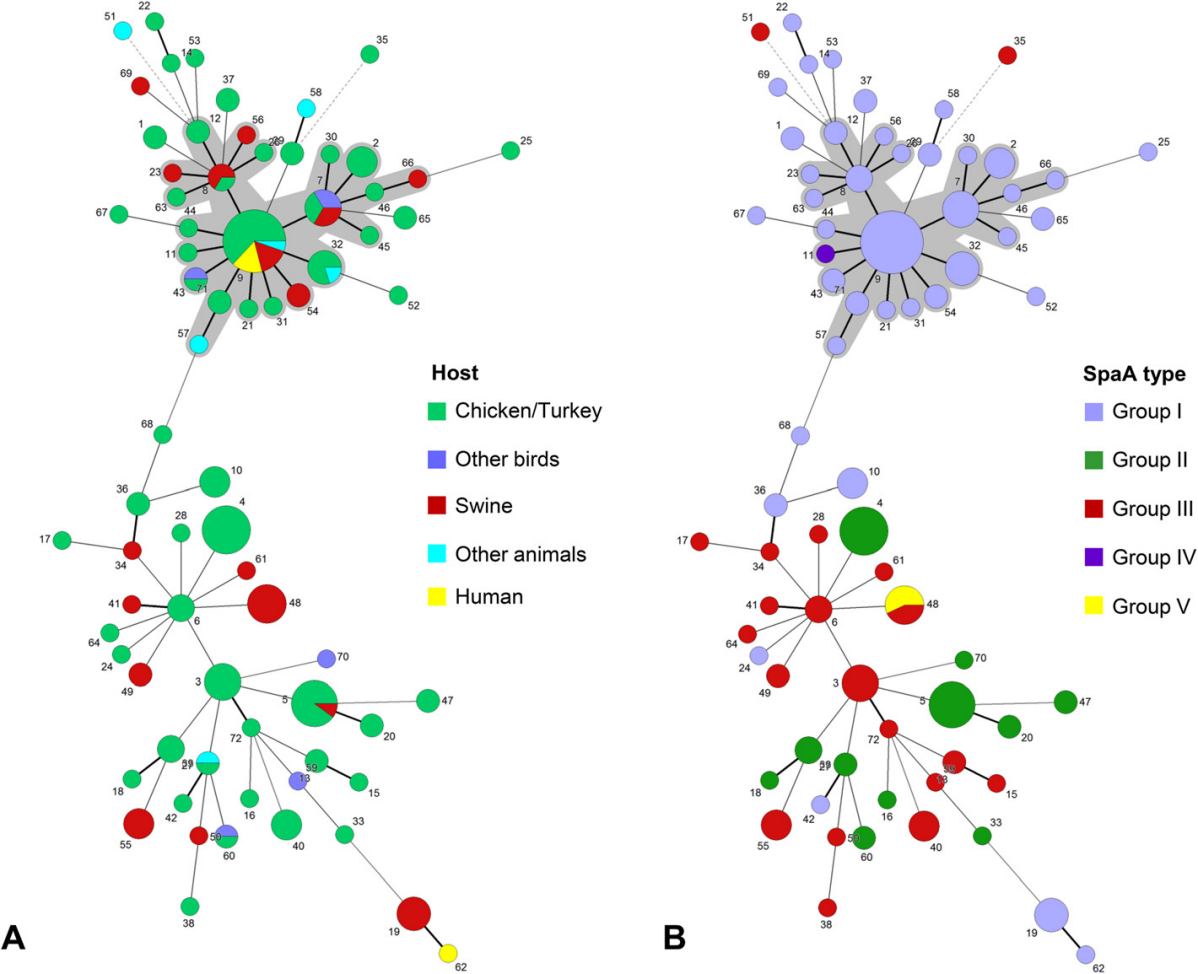
**Minimum spanning tree based on allele profiles of 165** ***E. rhusiopathiae*** **isolates and distribution of** **(A)** **host and (** **B** **) SpaA type** (for Groups I-V see Table 4). Sequence types (STs) are indicated by numbers and sequence type complexes are highlighted by grey shade.

Only one sequence type complex, i.e. a group of phylogenetic related strains was identified. ST complex 9 (STC9), named after its predicted founder, included 22 STs and accounted for 34.5% of the total collection of MLST-analysed strains. As depicted in Figure 2A, 40 avian and 17 mammalian *E. rhusiopathiae* strains were among the STC9 strains. In general, no host-specific or host-restricted sequence type was observed, as isolates from different hosts scattered around the various STs identified (Figure 2A). In addition, different countries shared isolates with identical STs, e.g. ST3 (isolates from Austria, Germany, Sweden), ST9 (Austria, Germany, USA), and ST19 (Estonia, Germany, USA), not suggesting any association between certain STs with distinct countries. However, due to a sample bias towards isolates from Germany, this should be verified by including a larger number of isolates from various countries.

### Pulsed-field gel electrophoresis and comparison of sequence types with PFGE clusters

**Figure 3:**
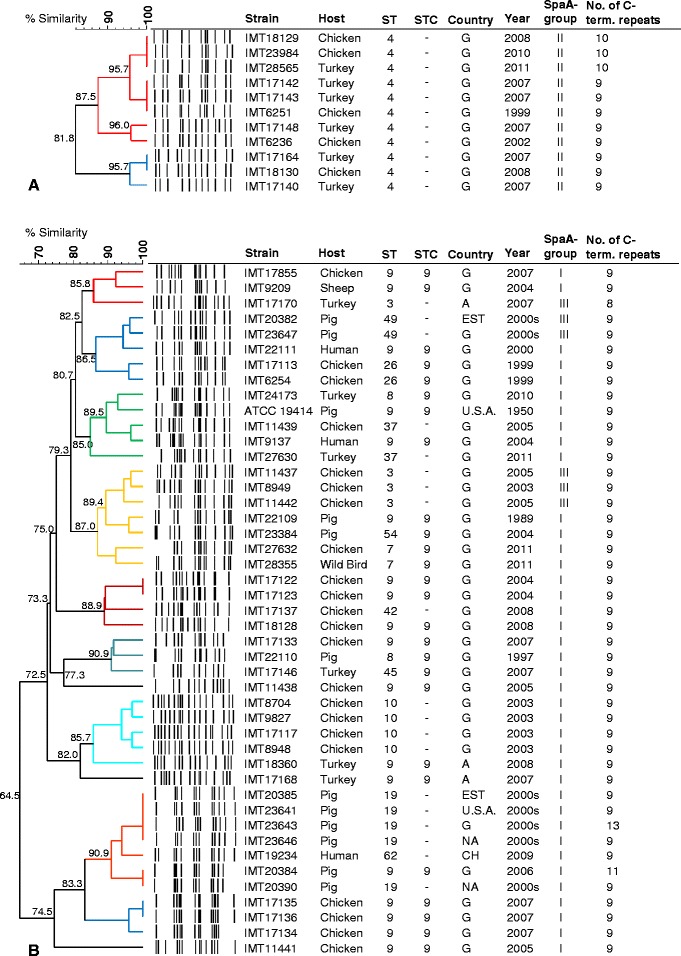
**Dendrogram for** ***E. rhusiopathiae*** **isolates based on PFGE banding patterns produced by SmaI restriction.** Illustrated are dendrograms for (**A**) 45 *E. rhusiopathiae* isolates of sequence type complex (STC) 9 and isolates of other STs clustering with ST9 strains and (**B**) 11 ST4 isolates that do not cluster with strains of other STs. Additional information on isolation year and country, host, SpaA-groups (based on amino acid substitutions) and the number of SpaA C-terminal repeats are provided in separate rows. PFGE clusters (85% cut-off) are indicated by random colours; singletons are left black.

Based on an 85% cut-off- value we identified 54 different PFGE clusters containing one (*n* = 17 singletons) to 13 strains among 165 *E. rhusiopathiae* isolates (Additional file 2). Identical macrorestriction profiles were only rarely observed which is consistent with our criteria for strain selection, i.e. exclusion of copy strains from the analysis. In contrast to PFGE, MLST revealed 72 different sequence types and, as expected, the grouping achieved by both methods revealed only partial congruence. The most homogenous macrorestriction profile was observed for strains assigned to ST4. All eleven ST4 strains, isolated from 1999 to 2011 from chickens and turkeys in Germany clustered exclusively together at a similarity value of 90.9% (Figure 3A). In contrast, strains of the most prominent sequence type ST9 were distributed among 12 different PFGE clusters, including three singletons, with an overall similarity of 64.5% (Figure 3B). These PFGE clusters frequently harboured strains of other sequence types as well, which is also the case for most of the other non-singleton PFGE clusters.

A calculation of the Simpsons index of discriminatory (*D*) with 95% confidence interval (CI_95%_) revealed that the ability of MLST to discriminate *E. rhusiopathiae* isolates (*D* = 0.973; CL_95%_ [0.963 to 0.983]) was similar to that of PFGE (*D* = 0.976; CL_95%_ [0.970 to 0.982]). However, as exemplified by the dendrograms shown in Figure 3 and in the Additional file 2, strain groups established by the band-based method PFGE and the sequence-based method MLST frequently differ in their composition.

### Virulence gene typing

Highly homogenous patterns of putative virulence genes were observed among the 165 *E. rhusiopathiae* isolates with 136 (82.4%) of them possessing all 16 investigated genes (Table 2). Another 24 (14.5%) isolates differed only by the absence of internalin gene *intI*. Five isolates lacked either *algI* (*n* = 4), or *dnaB* (*n* = 1), which might be associated with colonization and resistance to phagocytosis and with bacterial proliferation and adhesion, respectively [45]. None of the 16 genes investigated were present in the *E. inopinata* reference strain DSM 15511^T^, whereas *nanH* and *ewlA* were detected in the *E. tonsillarum* strain DSM 14972^T^ as well as in both field strains of *E. tonsillarum*.

### Spa typing

Sequence analysis of the amplified DNA fragments of *spa* genes revealed the consistent presence of *spaA* and the absence of *spaB* and *spaC*, respectively, among all 165 *E. rhusiopathiae* isolates. The size of *spaA* open reading frames ranged from 1761 to 2181 bp (encoding 587 to 727 amino acids). The signal sequences of 29 amino acids were conserved among the Spa proteins of 164 isolates (99.4%). Only one isolate from a duck (IMT28342; Acc.-No. KR606269) showed a mutation at nucleotide position 27 (A to G) and at amino acid position 10 (Lysin to Glutamic acid), respectively.

Sequences of the N-terminal hypervariable region of the *spaA* gene of field isolates and of *E. rhusiopathiae* serotype reference strains were compared with that of the virulent wild-type reference Fujisawa serotype 1a strain (see Table 4). Based on patterns of amino acid changes at eight different positions, the field isolates were divided into five groups as follows: (i) 87 isolates with isoleucine at position 55 (Ile-55), Asn-70, Asp-178, Asn-195, Ile-257 and Gln-303 classified as group I, (ii) 36 isolates with Ser-101 and Ile-257 classified as group II, (iii) 37 isolates with Ile-257 termed group III, (iv) one isolate with Ile-257 and Gln-303 classified as group IV, and (v) 4 isolates with Met-203 and Ile-257 termed group V (group I-V as specified in Table 4).

**Table 4:** Substitutions in amino acids in the N-terminal hypervariable region of the *spaA* gene and number of C-terminal tandem repeats in 165 *E. rhusiopathiae* field isolates and serotype reference strains compared with the corresponding sequence of *E. rhusiopathiae* Fujisawa strain^c^

*E. rhusiophatiae* strain or group (no. of isolates)		Substitutions in N-terminal nucleotides (amino acid position)^a,b^								No. of C-terminal tandem repeats
		Nucleotide (aa 55)	Nucleotide (aa 70)	Nucleotide (aa 101)	Nucleotide (aa 178)	Nucleotide (aa 195)	Nucleotide (aa 203)	Nucleotide (aa 257)	Nucleotide (aa 303)	
Fujisawa serotype 1a		GTA (Val)	AAA (Lys)	AAC (Asn)	GGT (Gly)	GAT (Asp)	ATT (Ile)	CTT Leu	GGG (Gly)	9
Field isolates (No.)	Group I (87)	ATA (Ile)	AAT (Asn)		GAT (Asp)	AAT (Asn)		ATT (Ile)	GAG (Gln)	8-11, 13
	Group II (36)			AGC (Ser)				ATT (Ile)		7-10
	Group III (37)							ATT (Ile)		8, 9
	Group IV (1)							ATT (Ile)	GAG (Gln)	8
	Group V (4)						ATG (Met)	ATT (Ile)		9
Serotype reference strains	Serotype 1a (Koganei) 5, 15	ATA (Ile)	AAT (Asn)		GAT (Asp)	AAT (Asn)		ATT (Ile)	GAG (Gln)	9
	Serotype 8, 17			AGC (Ser)				ATT (Ile)		9
	Serotype 1b, 9, 12, 16, N							ATT (Ile)		8, 9
	Serotype 2	ATA (Ile)	AAT (Asn)					ATT (Ile)		9

^a^Asp aspartic acid; Asn asparagine; Gln glutamine; Ile isoleucine; Leu leucine; Lys lysine; Met methionine; Val valine.

^b^Underlined character, nucleotide different from those at the same position; empty fields indicate absence of amino acid substitutions compared with the Fujisawa strain.

^c^Nucleotide sequences of *spaA* genes of Fujisawa strain and of *E. rhusiopathiae* serotype reference strains were obtained from GenBank (AB259652, strain Fujisawa, serotype 1a; AB024082, Koganei, serotype 1a; AB259653, 442/1E1, serotype 1b; AB259654, ATCC19414^T^, serotype 2; AB259655, Pècs 67, serotype 5; AB259656, Goda, serotype 8; AB290347, Kaparek, serotype 9; AB259657, Pècs 9, serotype 12; AB259658, Pècs 3597, serotype 15; AB259659, Tanzania, serotype 16; AB259660, 545, serotype 17; AB259661, MEW22, serotype N) and translated into amino acid sequences using RidomSeqSphere. Reference strains for other serotypes were not included as they chiefly harbor *spaB* genes (serotypes 4, 6, 11, 19, and 21) or a *spaC* gene (serotype 18), respectively [15].

Except for serotype 2 reference strain ATCC19414^T^, which revealed a unique pattern (Ile-55, Asn-70 and Ile-257) all other reference strains included (see Table 4) corresponded to one of the SpaA groups observed among the field isolates. None of the SpaA groups correlated with a certain host type (mixed avian and mammalian). As far as serotypes were available (*n* = 32; serotype 1a and 1b (*n* = 20); serotype 2 (*n* = 9), serotype N (*n* = 3), they also did not correlate with certain SpaA groups. In group I, we observed both serotype 1a/b and serotype 2 strains and within group III, strains of serotypes 1a/b, 2 and N were present (data not shown).

The number of C-terminal tandem repeats of similar 20-amino-acid sequences in the SpaA proteins of field isolates ranged from 7 to 13, corresponding to the different *spaA* nucleotide sequence lengths observed. The majority of strains (89.7%) harboured a domain with 9 repeats similar to that present in strain Fujisawa and in all SpaA-positive serotype reference strains, except for serotype 9 strain Kaparek (Table 4). The remaining field isolates showed either higher or lower number of repeats. One strain from a chicken with septicaemia was truncated by 40 amino acids at positions 502 and 534 and another seven strains lacked a 20 amino acids repeat either at position 534 or 558. Seven strains revealed 10 repeats and two porcine strains even showed 11 and 13 repetitions of similar 20-amino-acid sequences in the C-terminal part of their SpaA protein, respectively. A host-specific distribution of SpaA types was not observed. Portraying the different SpaA alleles against the phylogenetic background of the strains reveals a strong linkage of group I (amino acid changes Ile-55, Asn-70, Asp-178, Asn-195, Ile-257 and Gln-303 compared with Fujisawa strain) with ST complex 9 (Figure 2B). Group II (Ser-101, Ile-257) and group III isolates (Ile-257) occur in several STs which are not related to this major ST complex. Only in one case, different SpaA types occur in the same sequence type, namely ST48, which is made up by porcine isolates (Figure 2A).

### Nucleotide sequence accession numbers

Nucleotide sequence of *spaA* genes of 165 *E. rhusiopathiae* strains have been submitted to the GenBank database under accession nos. KR606141 – KR606305.

## Discussion

Although there is an increasing notion of severe outbreaks of Erysipelas in poultry [10,22,47], possibly related to the ban of conventional cages within EU from 2012 and the large changes of housing systems due to welfare demands for laying hens [9,10,12], only little is known about the molecular epidemiology of avian *E. rhusiopathiae* isolates. To overcome this, we investigated isolates of *E. rhusiopathiae*, mainly from poultry but also from mammalian hosts by a newly developed species-specific MLST scheme and in addition by more established methods such as PFGE, virulence genotyping and Spa typing.

The novel MLST scheme for *E. rhusiopathiae* is based on the identification of nucleotide polymorphisms of internal fragments from seven housekeeping genes involved in metabolic pathways of the bacterial pathogen. The target genes were chosen with a d_N_/d_S_ ratio of <1 to guarantee a very low degree of evolutionary pressure on the proteins and absence of a selective force on the respective gene locus. This ratio was calculated for other MLST schemes, recently developed e.g. for the bovine pathogen *Mannheimia haemolytica* [48] or for *Enterococcus faecalis*, which has been associated with amyloid arthropathy in chickens [49] as well. The lower number of alleles in the *E. rhusiopathiae* MLST scheme, which varies between 6 (*pta*) and 14 (*purA*) among 165 *E. rhusiopathiae* strains together with a diversity of index *D* of 0.69 (95% CI, 0.579 to 0.775), which is also lower than that observed for other schemes [48-51] might indicate a lower diversity as compared e.g. to the Gram negative pathogen *E. coli* or to the Gram positive species *Streptococcus gallolyticus*. However, this assumption should be treated cautiously as the MLST databases of these two species contain significantly more isolates, i.e. 7.500 isolates in case of *Escherichia coli* [52] and 292 isolates in case of *Sc. gallolyticus* [53]. Whether *E. rhusiopathiae* would still appear as a bacterial species with low diversity after including more isolates from different sources remains to be determined.

The distribution of the STs as presented in a minimum spanning tree (MST) shows accumulations of *E. rhusiopathiae* strains isolated from various animals in certain STs (Figure 2A). Even if there was a bias towards avian isolates and towards isolates from Germany in our sample material, there was no indication for host or geographic specificity. Strains from poultry and swine randomly scattered throughout the MSTree and commonly shared identical STs, such as ST9, ST8 and ST5. This might indicate that birds and pigs may be infected by phylogenetically similar strains either by a common reservoir or by direct or indirect transmission from one animal species to the other. A limited host specificity has also been suggested in another recent study, where marine *E. rhusiopathiae* isolates were shown to be capable of inducing classical skin lesions in pigs in a skin inoculation model [54]. However, looking deeper into the clonal nature of our strains by PFGE, those from poultry and pigs were commonly found in same PFGE clusters, but no identical pairs of isolates were observed between the two species. This supports a previous finding by Eriksson et al. who also didn’t observe a 100% band profile identity among any of the pig and poultry isolates albeit the strains were not separated into two distinct populations by PFGE analysis [22].

Notably, the most prominent ST observed in the present study – ST9 - includes human and animal strains. The grouping of porcine strain ATCC 19414^T^ and human strain IMT9137 in an identical sequence type and in the same PFGE cluster (89.9% similarity in banding pattern) indicates that these strains may be closely related and that a zoonotic potential of animals strains should be taken into consideration. Sporadic diseases in humans have been reported and these often occurred in farmers and in other persons whose work is closely related with contaminated animals, underlining that *E. rhusiopathiae* infection in humans is occupationally-related [2,55,56].

Overall, our MLST analysis distinguished 165 isolates into 72 sequence types (STs) and only partial congruence was observed to the grouping of strains into 58 PFGE clusters. MLST intends to portray the long term epidemiology and the evolutionary history of a bacterial pathogen based on a representative set of the bacterial core genome rather than to unravel disease outbreaks, at least not without additional typing methods [26]. In contrast, PFGE is a suitable tool to monitor short-term local outbreaks mainly by identifying clones [57].

In studies comparing MLST to PFGE e.g. among *Enterococcus faecalis*, *Staphylococcus aureus*, and *Vibrio cholerae* isolates, MLST has been found to have similar or greater discriminatory ability than PFGE [58-61]. In contrast, a study comparing MLST and PFGE data in *Escherichia coli* O157:H7, found that PFGE revealed a greater discriminatory ability than MLST did, which may be due to the highly clonal nature of this *E. coli* serotype [62]. In case of the Gram-negative pathogen *Pseudomonas aeruginosa* Johnson et al. found that MLST was better for detecting genetic relatedness, while PFGE was more discriminatory than MLST for determining genetic differences in this bacterial species and to confirm the a patient-to-patient transmission [63]. Nemoy et al. observed a greater discriminatory ability and reproducibility of MLST than PFGE for ESBL-producing *E. coli* [58]. They suggested MLST is suitable to a priori define genetically related bacterial strains, which might be very helpful for surveillance.

Our MLST data for the first time allow determining *E. rhusiopathiae* as a weakly clonal species as 165 isolates are dispersed among 72 different sequence types. The accumulation of a number of strains in ST complex 9 may indicate that this group of genetically related strains represents a particularly successful subpopulation. If this holds true after including more strains from different countries is uncertain. Likewise, possible reasons for this success or at least high prevalence of STC9 strains (e.g. high virulence, environmental survival) also need to be studied.

Interestingly, although serotypes for only 32 of our isolates were available (serotype 1a and 1b (*n* = 20); serotype 2 (*n* = 9); serotype N (*n* = 3)), these serotypes were not strictly linked with a certain sequence type or ST complex (data not shown). In addition, different serotypes were observed in identical STs, indicating that the O antigen encoding genetic structure may have, at least partly, evolved independently from the phylogenetic background of the strains, which has likewise been reported for other bacterial species, such as *E. coli* and *Bordetella* spp. [64,65]. No correlation between serotype and PFGE pattern could also be observed in a recent study including *E. rhusiopathiae* isolates from poultry, pigs, emus, and the poultry red mite *Dermanyssus gallinae* [22]. Here, the authors found that serotypes were randomly scattered throughout the dendrogram based on PFGE patterns and that isolates with the same PFGE pattern were of different serotypes in some cases. They concluded that serotyping may be less suitable for studies on epidemiological relationships between flocks.

Concerning the putative virulence genes investigated in our study, there was no congruence between one of these genes with a certain sequence type or PFGE cluster, which is primarily attributable to the frequent detection of the genes. Almost all 16 markers selected and putatively involved in pathogenesis, except for *intI* (85.5%), the Alginate-O-acetyltransferase gene *algI* (97.6%), and the attachment protein gene *dnaB* (99.4%) were regularly present in our isolates. According to the annotation of whole genome sequenced *E. rhusiopathiae* strains Fujisawa (GenBank AP012027) and SY1027 (GenBank CP005079) *intI* encodes an internalin-like protein. We observed 15% *intI*-negative isolates that occurred in 12 different STs, mostly from poultry and two isolates from sheep. But, as the function of this internalin-like protein has been deduced from amino acid sequence comparisons only, further molecular genetic studies and in vitro assays would have to be performed to verify its putative role in *E. rhusiopathiae* pathogenesis. Although most of the other genes were regularly present in our isolates, their expression might vary due to different body compartments or environmental factors. For some of these factors, such as the neuraminidase, a correlation between the produced amount of this enzyme, which may serve the nutritional requirements and play a role in adherence and antiphagocytic activity of the bacteria, and the virulence of strains has been reported [2,66]. It was not unexpected to detect the capsule biosynthesis locus in all our isolates. In previous studies capsule-negative mutants or the loss of capsule in general resulted in avirulent strains which did not resist phagocytosis by murine polymorphonuclear leukocytes and lost the capability of intracellular survival [2,40]. Thus, such strains would not have been able to cause severe disease in the various hosts.

This study was mainly focussed on poultry isolates, as we intended to provide a rational typing method to assess the clonal relatedness of these strains, particularly with respect to the selection of epidemiological relevant strains to be included in future vaccines. In this respect we also aimed to determine, whether the N-terminal hyper-variable region of the surface protein SpaA, which apart from SpaC, represents one of the most promising vaccine candidates [13-15] might show a co-evolution with the genomic backbone of *E. rhusiopathiae*. The exclusive finding of SpaA and the absence of SpaB and SpaC in our sample collection might be due the prevalence of certain serotypes among our strains. Our partial serotype data confirm what has been reported in previous studies, namely that the *spaA* gene usually, but not exclusively, occurs in strains belonging to serotype 1a, 1b, 2, 5, 8, 9, 12, 15, 16, 17 and type N, while *spaB* and *spaC* are mainly observed in *E. rhusiopathiae* serotypes 4, 6, 11, 19, and 21 and in serotype 18, respectively [3,13,14,17]. Ingebritson et al. reported that one strain may contain more than one Spa type and that these were not always confined to a certain serotype [3]. It is not likely that single isolates of our study harboured different Spa types as this would have become apparent due to ambiguous sequencing data.

Different authors have explored the variations in the N-terminal half of the *spaA* gene, particularly in the protective domain which is located at amino acid positions 30 to 413, and in the repeat domain which is located at amino acid positions 448 to 626 (in case of 8 to 10 tandem repeats) [14,67,68]. A broad collection of avian isolates and their *spa* gene has not been investigated so far, limiting our knowledge about the diversity of this major antigen considerably. By sequence analysis of the *spaA* gene of 165 clinical *E. rhusiopathiae* isolates, thereof 120 from birds, we found high sequence identity within the signal sequence region, located at the most N-terminal part of the Spa protein, corroborating the findings of To and Nagai and supporting the idea that Spa proteins may cross bacterial cell membranes and eventually be secreted from the bacterial cell by using a secretion machinery commonly present in *E. rhusiopathiae* strains [14]. Although most of our isolates (89.7%) revealed nine C-terminal tandem repeats in their SpaA protein, which is also common to most of the serotype reference strains investigated by To and Nagai and listed in Table 4 [14], the diversity in number was quite high with 7 to 13 tandem repeats and has not been reported before. As this repeat domain has been suggested to function as an anchor for binding Spa proteins tightly to the bacterial surface it remains to be determined, e.g. by in vitro adhesion experiments, if this binding might perhaps be influenced by the number of repeats [13,14,68]. Regarding the amino acid substitutions in the N-terminal protective part of the Spa protein we detected five different variants (group I-V) that occurred independently of the original host species and have been described at least in part in previous publications on porcine isolates as well. Among swine isolates from Japan, Uchiyama et al. observed three groups, looking at substitutions at amino acid positions 195, 203 and 257 [17]. These positions were also part of our investigations, while we additionally included another 6 amino acid substitutions resulting in a finer resolution of the groups. Thus, their group 2 resembles our groups II-IV; group 1 matches with our group V; and group 3 was not detected among our isolates at all. In that study, more than half of the 34 porcine strains showed the Met-203 variant in combination with Ile-257 (group 1 according to Uchiyama et al. [17]), corresponding to group 2 recently determined among another collection of isolates from Japan [69]. To et al. [68] found that 95% of their overall 80 pig isolates carried the Met-203 variant of the *spaA*, while the same variant accounted for 55.6% of the isolates included by Uchiyama et al. [17]. Notably, we observed this variant only very infrequently (2.4%) among our isolates. In the present strain collection we rather detected SpaA variants (group I, 52.7%; group II, 21.8%; group III, 22.4%) that have been described for different serotype reference strains before (Table 4) [14]. Of note, the distribution of *spaA* variants demonstrated to be irrespective of the original host of the strains, i.e. avian or mammalian. Whether the current geographic linkage of the Met-203 variant to Japan remains after studying more isolates from different countries is uncertain [17,69]. Uchiyama et al. could show that isolates with Met-203 were highly pathogenic in mice with LD_50_ values between 0.45 and 1.45 CFU/mouse, but comparative in vivo data with isolates representing other *spaA* groups were not provided [17]. The same authors showed that the commercial vaccine Koganei 65–0.15, which lacks this amino acid substitution, was still protective against an experimental infection of pigs with a Met-203 variant. Thus, it was concluded that the respective protein region might not be that essential for protection against *E. rhusiopathiae* infections [17].

Our analysis was based on a comparison with the Fujisawa serotype 1a strain to allow for a comparison with already published data, that also used this strain as reference [17,67,69]. As we did not have access to one of the vaccine strains (inactivated whole cell bacteria) currently used in the field in Germany for protection of swine against Erysipelas, a comparison of our strain collection with these strains still has to be done. Moreover, targeted virulence assays including the most prominent sequence types and different *spaA* variants might provide important insight into the impact of a strain’s genetic background and its potential to cause disease.

In summary, with the development of the MLST scheme a highly reproducible epidemiological tool to study *E. rhusiopathiae* strains has been established that allows for long-term global studies to assess the evolutionary history and genetic relatedness of isolates belonging to this bacterial pathogen. Clinical isolates were found to be highly clonal on the one hand (ST complex 9) but also scattered around the entire population of the selected collection. Applying this MLST technique clearly unravelled a common evolutionary origin of isolates obtained both from avian and mammalian hosts indicating an interspecies transmission and/or common reservoir and a zoonotic potential. Combined data of MLST, PFGE and SpaA typing provided a good basis for assessing the clonal diversity and molecular make-up of *E. rhusiopathiae*. This might be very advantageous in several aspects, including the (i) selection of epidemiological relevant vaccine candidates (ii) estimation of the pathogenic relevance of single putative virulence genes and isolates in future in vitro and in vivo experiments, and (iii) the assessment of the zoonotic risk of the pathogen.

## Additional files

Additional file 1:
**Allele profiles, multilocus sequence types (STs) and Spa types of 165** ***E. rhusiopathiae*** **isolates determined in this study.** This table provides all relevant data of the 165 *E. rhusiopathiae* isolates typed by multilocus sequence typing in the present study. The data listed include allele types, sequence types (ST), ST complexes (if any), grouping of SpaA protein sequences based on N-terminal sequences and number of C-terminal repeats of the isolates. In addition, the year of isolation as well as host origin and country of isolation are provided for each of the isolate. Additional file 2:
**Dendrogram for 165 field isolates from birds and mammals and the type strain of** ***Erysipelothrix rhusiopathiae*** **ATCC 19414** ^**T**^ **, based on pulsed-field gel electrophoresis macrorestriction patterns generated by digestion with SmaI restriction endonuclease.** This table provides PFGE banding patterns of 165 *E. rhusiopathiae* isolates with their sequence types (STs), ST complexes (if applicable), and epidemiological background (year, country, and host of isolation). Abbreviations: A = Austria, CH = Switzerland, DK = Denmark, EST = Republic of Estonia, G = Germany, NA = unknown, S = Sweden, USA = United States of America.
