# Supplementary material for: A combinational approach of multilocus sequence typing and other molecular typing methods in unravelling the epidemiology of Erysipelothrix rhusiopathiae strains from poultry and mammals
Source: Vet Res. 2015 Jul 21;46(1):84. doi: 10.1186/s13567-015-0216-x (PMC4509749; doi:10.1186/s13567-015-0216-x)
Supplement: Additional file 1: — Allele profiles, multilocus sequence types (STs) and Spa types of 165 E. rhusiopathiae isolates determined in this study. This table provides all relevant data of the 165 E. rhusiopathiae isolates typed by multilocus sequence typing in the present study. The data listed include allele types, sequence types (ST), ST complexes (if any), grouping of SpaA protein sequences based on N-terminal sequences and number of C-terminal repeats of the isolates. In addition, the year of isolation as well as host origin and country of isolation are provided for each of the isolate. [file 13567_2015_216_MOESM1_ESM.docx]

| **Strain** | **ST** | **STC** | ***gpsA*** | ***recA*** | ***purA*** | ***pta*** | ***prsA*** | ***alK*** | ***ldhA*** | **SpaA-N-term.  group^a^** | **SpaA-C-term. repeats^b^** | **Year of isolation** | **Host** | **Country** |
| --- | --- | --- | --- | --- | --- | --- | --- | --- | --- | --- | --- | --- | --- | --- |
| ATCC 19414^T^ | 9 | 9 | 1 | 1 | 1 | 1 | 1 | 1 | 1 | I | 9 | 1950 | Pig | USA |
| IMT11437 | 3 | - | 2 | 4 | 3 | 2 | 2 | 2 | 2 | III | 9 | 2005 | Chicken | G |
| IMT11438 | 9 | 9 | 1 | 1 | 1 | 1 | 1 | 1 | 1 | I | 9 | 2005 | Chicken | G |
| IMT11439 | 37 | - | 6 | 1 | 1 | 2 | 1 | 4 | 1 | I | 9 | 2005 | Chicken | G |
| IMT11441 | 9 | 9 | 1 | 1 | 1 | 1 | 1 | 1 | 1 | I | 9 | 2005 | Chicken | G |
| IMT11442 | 3 | - | 2 | 4 | 3 | 2 | 2 | 2 | 2 | III | 9 | 2005 | Chicken | G |
| IMT11443 | 5 | - | 2 | 4 | 4 | 2 | 2 | 2 | 3 | II | 9 | 2005 | Chicken | G |
| IMT11444 | 11 | 9 | 1 | 1 | 1 | 1 | 1 | 1 | 3 | IV | 8 | 2005 | Chicken | G |
| IMT11445 | 25 | - | 5 | 3 | 2 | 5 | 4 | 1 | 1 | I | 9 | 2005 | Chicken | S |
| IMT13536 | 8 | 9 | 1 | 1 | 1 | 1 | 1 | 4 | 1 | I | 9 | 2007 | Pig | G |
| IMT14192 | 27 | - | 2 | 5 | 3 | 2 | 2 | 2 | 3 | II | 9 | 2007 | Chicken | G |
| IMT17113 | 26 | 9 | 1 | 4 | 1 | 1 | 1 | 5 | 1 | I | 9 | 1999 | Chicken | G |
| IMT17115 | 31 | 9 | 1 | 1 | 1 | 1 | 1 | 1 | 6 | I | 9 | 1999 | Chicken | G |
| IMT17117 | 10 | - | 1 | 1 | 6 | 2 | 2 | 2 | 2 | I | 9 | 2003 | Chicken | G |
| IMT17119 | 5 | - | 2 | 4 | 4 | 2 | 2 | 2 | 3 | II | 9 | 2003 | Chicken | G |
| IMT17120 | 38 | - | 2 | 5 | 10 | 2 | 4 | 5 | 3 | III | 9 | 2003 | Chicken | G |
| IMT17121 | 5 | - | 2 | 4 | 4 | 2 | 2 | 2 | 3 | II | 8 | 2004 | Chicken | G |
| IMT17122 | 9 | 9 | 1 | 1 | 1 | 1 | 1 | 1 | 1 | I | 9 | 2004 | Chicken | G |
| IMT17123 | 9 | 9 | 1 | 1 | 1 | 1 | 1 | 1 | 1 | I | 9 | 2004 | Chicken | G |
| IMT17124 | 39 | - | 2 | 5 | 3 | 2 | 2 | 5 | 2 | II | 9 | 2004 | Chicken | G |
| IMT17125 | 7 | 9 | 1 | 3 | 1 | 1 | 1 | 1 | 1 | I | 9 | 2004 | Chicken | G |
| IMT17129 | 6 | - | 2 | 2 | 2 | 2 | 2 | 2 | 2 | II | 9 | 2006 | Chicken | DK |
| IMT17130 | 27 | - | 2 | 5 | 3 | 2 | 2 | 2 | 3 | I | 10 | 2006 | Chicken | G |
| IMT17131 | 32 | 9 | 5 | 1 | 1 | 1 | 1 | 1 | 1 | I | 9 | 2006 | Chicken | G |
| IMT17132 | 32 | 9 | 5 | 1 | 1 | 1 | 1 | 1 | 1 | I | 9 | 2001 | Chicken | G |
| IMT17133 | 9 | 9 | 1 | 1 | 1 | 1 | 1 | 1 | 1 | I | 9 | 2007 | Chicken | G |
| IMT17134 | 9 | 9 | 1 | 1 | 1 | 1 | 1 | 1 | 1 | I | 9 | 2007 | Chicken | G |
| IMT17135 | 9 | 9 | 1 | 1 | 1 | 1 | 1 | 1 | 1 | I | 9 | 2007 | Chicken | G |
| IMT17136 | 9 | 9 | 1 | 1 | 1 | 1 | 1 | 1 | 1 | I | 9 | 2007 | Chicken | G |
| IMT17137 | 42 | - | 2 | 5 | 3 | 2 | 2 | 8 | 2 | I | 9 | 2008 | Chicken | G |
| IMT17138 | 2 | 9 | 1 | 3 | 1 | 3 | 1 | 1 | 1 | I | 9 | 2007 | Turkey | G |
| IMT17139 | 5 | - | 2 | 4 | 4 | 2 | 2 | 2 | 3 | II | 8 | 2008 | Chicken | G |
| IMT17140 | 4 | - | 2 | 4 | 2 | 2 | 2 | 5 | 2 | II | 9 | 2007 | Turkey | G |
| IMT17142 | 4 | - | 2 | 4 | 2 | 2 | 2 | 5 | 2 | II | 9 | 2007 | Turkey | G |
| IMT17143 | 4 | - | 2 | 4 | 2 | 2 | 2 | 5 | 2 | II | 9 | 2007 | Turkey | G |
| IMT17144 | 12 | 9 | 1 | 2 | 1 | 1 | 1 | 4 | 1 | I | 9 | 2007 | Turkey | G |
| IMT17146 | 45 | 9 | 1 | 3 | 1 | 1 | 1 | 1 | 1 | I | 9 | 2007 | Turkey | G |
| IMT17147 | 12 | 9 | 1 | 2 | 1 | 1 | 1 | 4 | 1 | I | 9 | 2007 | Turkey | G |
| IMT17148 | 4 | - | 2 | 4 | 2 | 2 | 2 | 5 | 2 | II | 9 | 2007 | Turkey | G |
| IMT17149 | 13 | - | 2 | 4 | 2 | 2 | 4 | 6 | 2 | III | 9 | 2007 | Turkey | G |
| IMT17152 | 33 | - | 2 | 1 | 3 | 2 | 1 | 1 | 2 | II | 9 | 2007 | Turkey | G |
| IMT17154 | 14 | - | 3 | 2 | 1 | 1 | 1 | 3 | 1 | I | 9 | 2007 | Turkey | G |
| IMT17155 | 2 | 9 | 1 | 3 | 1 | 3 | 1 | 1 | 1 | I | 9 | 2007 | Turkey | G |
| IMT17157 | 15 | - | 2 | 4 | 7 | 2 | 4 | 6 | 2 | III | 9 | 2007 | Turkey | G |
| IMT17160 | 2 | 9 | 1 | 3 | 1 | 3 | 1 | 1 | 1 | I | 9 | 2007 | Turkey | G |
| IMT17161 | 28 | - | 2 | 5 | 6 | 2 | 2 | 2 | 2 | III | 9 | 2007 | Turkey | G |
| IMT17162 | 13 | - | 2 | 4 | 2 | 2 | 4 | 6 | 2 | III | 9 | 2007 | Turkey | G |
| IMT17164 | 4 | - | 2 | 4 | 2 | 2 | 2 | 5 | 2 | II | 9 | 2007 | Turkey | G |
| IMT17166 | 29 | 9 | 1 | 6 | 1 | 1 | 1 | 4 | 1 | I | 9 | 2007 | Turkey | A |
| IMT17168 | 9 | 9 | 1 | 1 | 1 | 1 | 1 | 1 | 1 | I | 9 | 2007 | Turkey | A |
| IMT17170 | 3 | - | 2 | 4 | 3 | 2 | 2 | 2 | 2 | III | 8 | 2007 | Turkey | A |
| IMT17852 | 6 | - | 2 | 2 | 2 | 2 | 2 | 2 | 2 | III | 9 | 2007 | Chicken | DK |
| IMT17853 | 30 | 9 | 1 | 3 | 11 | 1 | 1 | 1 | 1 | I | 9 | 2007 | Chicken | G |
| IMT17854 | 6 | - | 2 | 2 | 2 | 2 | 2 | 2 | 2 | III | 9 | 2007 | Chicken | DK |
| IMT17855 | 9 | 9 | 1 | 1 | 1 | 1 | 1 | 1 | 1 | I | 9 | 2007 | Chicken | G |
| IMT18127 | 43 | 9 | 1 | 1 | 1 | 1 | 3 | 1 | 1 | I | 9 | 2008 | Chicken | G |
| IMT18128 | 9 | 9 | 1 | 1 | 1 | 1 | 1 | 1 | 1 | I | 9 | 2008 | Chicken | G |
| IMT18129 | 4 | - | 2 | 4 | 2 | 2 | 2 | 5 | 2 | II | 10 | 2008 | Chicken | G |
| IMT18130 | 4 | - | 2 | 4 | 2 | 2 | 2 | 5 | 2 | II | 9 | 2008 | Chicken | G |
| IMT18232 | 48 | - | 2 | 2 | 2 | 2 | 5 | 1 | 2 | V | 9 | 2009 | Pig | G |
| IMT18358 | 36 | - | 1 | 2 | 6 | 2 | 2 | 5 | 2 | I | 9 | 2005 | Turkey | A |
| IMT18360 | 9 | 9 | 1 | 1 | 1 | 1 | 1 | 1 | 1 | I | 9 | 2008 | Turkey | A |
| IMT18361 | 40 | - | 2 | 4 | 1 | 7 | 4 | 2 | 3 | III | 9 | 2008 | Turkey | A |
| IMT18362 | 16 | - | 2 | 5 | 8 | 2 | 4 | 2 | 2 | II | 9 | 2008 | Turkey | A |
| IMT18363 | 17 | - | 2 | 1 | 6 | 1 | 2 | 5 | 2 | III | 9 | 2008 | Turkey | A |
| IMT18364 | 18 | - | 2 | 5 | 5 | 2 | 2 | 2 | 3 | II | 9 | 2008 | Turkey | A |
| IMT18365 | 47 | - | 2 | 4 | 2 | 8 | 2 | 6 | 3 | II | 9 | 2008 | Turkey | A |
| IMT18366 | 44 | 9 | 1 | 1 | 1 | 1 | 2 | 1 | 1 | I | 9 | 2008 | Turkey | A |
| IMT19234 | 62 | - | 2 | 4 | 9 | 1 | 1 | 12 | 2 | I | 9 | 2009 | Human | CH |
| IMT20382 | 49 | - | 2 | 2 | 14 | 2 | 2 | 2 | 8 | III | 9 | 2000s | Pig | EST |
| IMT20383 | 7 | 9 | 1 | 3 | 1 | 1 | 1 | 1 | 1 | I | 9 | 2000s | Pig | NA |
| IMT20384 | 9 | 9 | 1 | 1 | 1 | 1 | 1 | 1 | 1 | I | 11 | 2000s | Pig | G |
| IMT20385 | 19 | - | 2 | 4 | 9 | 1 | 1 | 1 | 2 | I | 9 | 2000s | Pig | EST |
| IMT20386 | 34 | - | 2 | 2 | 6 | 2 | 2 | 5 | 2 | III | 9 | 2000s | Pig | NA |
| IMT20388 | 41 | - | 2 | 2 | 2 | 2 | 4 | 2 | 2 | III | 9 | 2000s | Pig | NA |
| IMT20389 | 48 | - | 2 | 2 | 2 | 2 | 5 | 1 | 2 | III | 9 | 2000s | Pig | NA |
| IMT20390 | 19 | - | 2 | 4 | 9 | 1 | 1 | 1 | 2 | I | 9 | 2000s | Pig | NA |
| IMT20391 | 48 | - | 2 | 2 | 2 | 2 | 5 | 1 | 2 | III | 9 | 2000s | Pig | NA |
| IMT20877 | 63 | 9 | 1 | 9 | 1 | 1 | 1 | 4 | 1 | I | 9 | 2010 | Chicken | G |
| IMT22109 | 9 | 9 | 1 | 1 | 1 | 1 | 1 | 1 | 1 | I | 9 | 1989 | Pig | G |
| IMT22110 | 8 | 9 | 1 | 1 | 1 | 1 | 1 | 4 | 1 | I | 9 | 1997 | Pig | G |
| IMT22111 | 9 | 9 | 1 | 1 | 1 | 1 | 1 | 1 | 1 | I | 9 | 2000 | Human | G |
| IMT22113 | 50 | - | 2 | 5 | 2 | 2 | 4 | 5 | 2 | III | 9 | 2007 | Pig | G |
| IMT22114 | 51 | - | 1 | 2 | 2 | 1 | 4 | 10 | 10 | III | 9 | 2007 | Mouflon | G |
| IMT22115 | 52 | - | 5 | 8 | 1 | 1 | 1 | 1 | 9 | I | 9 | 2007 | Turkey | G |
| IMT22116 | 54 | 9 | 7 | 1 | 1 | 1 | 1 | 1 | 1 | I | 9 | 2008 | Pig | G |
| IMT22118 | 43 | 9 | 1 | 1 | 1 | 1 | 3 | 1 | 1 | I | 9 | 2009 | Pheasant | G |
| IMT23383 | 56 | 9 | 1 | 1 | 1 | 1 | 6 | 4 | 1 | I | 9 | 2002 | Pig | G |
| IMT23384 | 54 | 9 | 7 | 1 | 1 | 1 | 1 | 1 | 1 | I | 9 | 2004 | Pig | G |
| IMT23385 | 57 | 9 | 1 | 2 | 1 | 1 | 1 | 2 | 1 | I | 9 | 2001 | Ape | G |
| IMT23386 | 40 | - | 2 | 4 | 1 | 7 | 4 | 2 | 3 | III | 9 | 2010 | Turkey | G |
| IMT23431 | 40 | - | 2 | 4 | 1 | 7 | 4 | 2 | 3 | III | 9 | 2010 | Turkey | G |
| IMT23636 | 55 | - | 2 | 2 | 3 | 2 | 4 | 2 | 3 | III | 9 | 2000s | Pig | G |
| IMT23637 | 55 | - | 2 | 2 | 3 | 2 | 4 | 2 | 3 | III | 9 | 2000s | Pig | G |
| IMT23638 | 48 | - | 2 | 2 | 2 | 2 | 5 | 1 | 2 | V | 9 | 2000s | Pig | G |
| IMT23639 | 7 | 9 | 1 | 3 | 1 | 1 | 1 | 1 | 1 | I | 9 | 2000s | Pig | G |
| IMT23640 | 55 | - | 2 | 2 | 3 | 2 | 4 | 2 | 3 | III | 9 | 2000s | Pig | G |
| IMT23641 | 19 | - | 2 | 4 | 9 | 1 | 1 | 1 | 2 | I | 9 | 2000s | Pig | USA |
| IMT23642 | 48 | - | 2 | 2 | 2 | 2 | 5 | 1 | 2 | V | 9 | 2000s | Pig | G |
| IMT23643 | 19 | - | 2 | 4 | 9 | 1 | 1 | 1 | 2 | I | 13 | 2000s | Pig | G |
| IMT23644 | 48 | - | 2 | 2 | 2 | 2 | 5 | 1 | 2 | III | 9 | 2000s | Pig | G |
| IMT23645 | 55 | - | 2 | 2 | 3 | 2 | 4 | 2 | 3 | III | 9 | 2000s | Pig | G |
| IMT23646 | 19 | - | 2 | 4 | 9 | 1 | 1 | 1 | 2 | I | 9 | 2000s | Pig | NA |
| IMT23647 | 49 | - | 2 | 2 | 14 | 2 | 2 | 2 | 8 | III | 9 | 2000s | Pig | G |
| IMT23660 | 48 | - | 2 | 2 | 2 | 2 | 5 | 1 | 2 | V | 9 | 2007 | Pig | G |
| IMT23661 | 32 | 9 | 5 | 1 | 1 | 1 | 1 | 1 | 1 | I | 9 | 2004 | Chicken | G |
| IMT23662 | 32 | 9 | 5 | 1 | 1 | 1 | 1 | 1 | 1 | I | 9 | 2004 | Chicken | G |
| IMT23666 | 58 | - | 1 | 4 | 1 | 9 | 1 | 5 | 1 | I | 9 | 2005 | Sheep | G |
| IMT23667 | 39 | - | 2 | 5 | 3 | 2 | 2 | 5 | 2 | II | 9 | 2009 | Sheep | G |
| IMT23668 | 59 | - | 2 | 1 | 3 | 2 | 4 | 4 | 2 | III | 9 | 2010 | Duck | G |
| IMT23846 | 32 | 9 | 5 | 1 | 1 | 1 | 1 | 1 | 1 | I | 9 | 2006 | Hare | G |
| IMT23848 | 61 | - | 2 | 5 | 2 | 2 | 2 | 11 | 2 | III | 9 | 2007 | Pig | G |
| IMT23849 | 60 | - | 2 | 1 | 3 | 2 | 2 | 5 | 11 | II | 10 | 2009 | Stork | G |
| IMT23851 | 40 | - | 2 | 4 | 1 | 7 | 4 | 2 | 3 | III | 9 | 2010 | Turkey | G |
| IMT23888 | 5 | - | 2 | 4 | 4 | 2 | 2 | 2 | 3 | II | 9 | 2008 | Pig | G |
| IMT23984 | 4 | - | 2 | 4 | 2 | 2 | 2 | 5 | 2 | II | 10 | 2010 | Chicken | G |
| IMT24173 | 8 | 9 | 1 | 1 | 1 | 1 | 1 | 4 | 1 | I | 9 | 2010 | Turkey | G |
| IMT24999 | 64 | - | 2 | 2 | 6 | 2 | 2 | 2 | 3 | III | 9 | 2010 | Turkey | G |
| IMT25000 | 65 | - | 8 | 3 | 2 | 1 | 1 | 1 | 1 | I | 9 | 2010 | Turkey | G |
| IMT25001 | 65 | - | 8 | 3 | 2 | 1 | 1 | 1 | 1 | I | 9 | 2011 | Turkey | G |
| IMT25299 | 67 | - | 5 | 1 | 2 | 1 | 2 | 1 | 1 | I | 9 | 2011 | Chicken | G |
| IMT25300 | 27 | - | 2 | 5 | 3 | 2 | 2 | 2 | 3 | II | 9 | 2011 | Chicken | G |
| IMT25301 | 68 | - | 1 | 2 | 6 | 1 | 2 | 2 | 2 | I | 9 | 2010 | Chicken | G |
| IMT25575 | 66 | 9 | 1 | 3 | 13 | 1 | 4 | 1 | 1 | I | 9 | 2011 | Pig | G |
| IMT26577 | 47 | - | 2 | 4 | 2 | 8 | 2 | 6 | 3 | II | 9 | 2011 | Turkey | G |
| IMT26588 | 69 | - | 5 | 2 | 6 | 1 | 1 | 4 | 1 | I | 9 | 2011 | Pig | G |
| IMT27630 | 37 | - | 6 | 1 | 1 | 2 | 1 | 4 | 1 | I | 9 | 2011 | Turkey | G |
| IMT27631 | 60 | - | 2 | 1 | 3 | 2 | 2 | 5 | 11 | II | 7 | 2011 | Chicken | G |
| IMT27632 | 7 | 9 | 1 | 3 | 1 | 1 | 1 | 1 | 1 | I | 9 | 2011 | Chicken | G |
| IMT28342 | 70 | - | 9 | 3 | 3 | 2 | 2 | 13 | 2 | II | 9 | 2011 | Duck | G |
| IMT28355 | 7 | 9 | 1 | 3 | 1 | 1 | 1 | 1 | 1 | I | 9 | 2011 | Black-headed Gonolek | G |
| IMT28564 | 72 | - | 2 | 4 | 3 | 2 | 4 | 2 | 2 | III | 9 | 2011 | Turkey | G |
| IMT28565 | 4 | - | 2 | 4 | 2 | 2 | 2 | 5 | 2 | II | 10 | 2011 | Turkey | G |
| IMT28566 | 71 | 9 | 1 | 1 | 1 | 1 | 1 | 2 | 1 | I | 10 | 2011 | Turkey | G |
| IMT28568 | 71 | 9 | 1 | 1 | 1 | 1 | 1 | 2 | 1 | I | 10 | 2012 | Turkey | G |
| IMT4530 | 35 | - | 1 | 7 | 12 | 6 | 1 | 5 | 7 | III | 9 | 2000 | Chicken | G |
| IMT4544 | 7 | 9 | 1 | 3 | 1 | 1 | 1 | 1 | 1 | I | 8 | 1999 | Parrot | G |
| IMT4996 | 20 | - | 2 | 4 | 4 | 2 | 2 | 2 | 5 | II | 8 | 2000 | Chicken | G |
| IMT6233 | 5 | - | 2 | 4 | 4 | 2 | 2 | 2 | 3 | II | 9 | 2001 | Chicken | G |
| IMT6234 | 5 | - | 2 | 4 | 4 | 2 | 2 | 2 | 3 | II | 9 | 2001 | Chicken | G |
| IMT6235 | 1 | - | 1 | 3 | 1 | 1 | 1 | 4 | 4 | I | 9 | 2002 | Chicken | G |
| IMT6236 | 4 | - | 2 | 4 | 2 | 2 | 2 | 5 | 2 | II | 9 | 2002 | Chicken | G |
| IMT6237 | 1 | - | 1 | 3 | 1 | 1 | 1 | 4 | 4 | I | 9 | 2002 | Chicken | G |
| IMT6238 | 3 | - | 2 | 4 | 3 | 2 | 2 | 2 | 2 | III | 9 | 2002 | Chicken | G |
| IMT6241 | 20 | - | 2 | 4 | 4 | 2 | 2 | 2 | 5 | II | 8 | 2000 | Chicken | G |
| IMT6242 | 53 | - | 6 | 2 | 1 | 1 | 1 | 9 | 1 | I | 9 | 2000 | Chicken | G |
| IMT6243 | 5 | - | 2 | 4 | 4 | 2 | 2 | 2 | 3 | II | 9 | 2002 | Chicken | G |
| IMT6244 | 21 | 9 | 1 | 1 | 1 | 1 | 1 | 7 | 1 | I | 9 | 2001 | Turkey | G |
| IMT6245 | 3 | - | 2 | 4 | 3 | 2 | 2 | 2 | 2 | III | 9 | 2002 | Chicken | S |
| IMT6247 | 22 | - | 3 | 2 | 1 | 4 | 1 | 3 | 1 | I | 9 | 2001 | Chicken | S |
| IMT6248 | 36 | - | 1 | 2 | 6 | 2 | 2 | 5 | 2 | I | 9 | 2001 | Chicken | G |
| IMT6250 | 5 | - | 2 | 4 | 4 | 2 | 2 | 2 | 3 | II | 9 | 1998 | Chicken | G |
| IMT6251 | 4 | - | 2 | 4 | 2 | 2 | 2 | 5 | 2 | II | 9 | 1999 | Chicken | G |
| IMT6252 | 2 | 9 | 1 | 3 | 1 | 3 | 1 | 1 | 1 | I | 9 | 1999 | Chicken | G |
| IMT6253 | 5 | - | 2 | 4 | 4 | 2 | 2 | 2 | 3 | II | 9 | 1999 | Chicken | G |
| IMT6254 | 26 | 9 | 1 | 4 | 1 | 1 | 1 | 5 | 1 | I | 9 | 1999 | Chicken | G |
| IMT6742 | 23 | 9 | 1 | 1 | 1 | 1 | 3 | 4 | 1 | I | 9 | 2003 | Pig | G |
| IMT8704 | 10 | - | 1 | 1 | 6 | 2 | 2 | 2 | 2 | I | 9 | 2003 | Chicken | G |
| IMT8705 | 46 | 9 | 1 | 3 | 13 | 1 | 1 | 1 | 1 | I | 9 | 2003 | Chicken | G |
| IMT8948 | 10 | - | 1 | 1 | 6 | 2 | 2 | 2 | 2 | I | 9 | 2003 | Chicken | G |
| IMT8949 | 3 | - | 2 | 4 | 3 | 2 | 2 | 2 | 2 | III | 9 | 2003 | Chicken | G |
| IMT9137 | 9 | 9 | 1 | 1 | 1 | 1 | 1 | 1 | 1 | I | 9 | 2004 | Human | G |
| IMT9209 | 9 | 9 | 1 | 1 | 1 | 1 | 1 | 1 | 1 | I | 9 | 2004 | Sheep | G |
| IMT9825 | 24 | - | 4 | 2 | 6 | 2 | 2 | 2 | 2 | I | 9 | 2004 | Chicken | G |
| IMT9827 | 10 | - | 1 | 1 | 6 | 2 | 2 | 2 | 2 | I | 9 | 2003 | Chicken | G |

A = Austria, CH = Switzerland, G = Germany, DK = Denmark, EST = Republic of Estonia, S = Sweden, USA = United States of America; NA = not applicable.

^a^ Groups I-V refer to the amino acid substitutions in the N-terminal region of the SpaA protein as given in Table 4 of the main manuscript.

^b^ The numbers refer to the number of 20-amino acid repeats in the C-terminal tandem repeat region of the SpaA protein.
