# Supplementary figures and images for: A combinational approach of multilocus sequence typing and other molecular typing methods in unravelling the epidemiology of Erysipelothrix rhusiopathiae strains from poultry and mammals
Source: Vet Res. 2015 Jul 21;46(1):84. doi: 10.1186/s13567-015-0216-x (PMC4509749; doi:10.1186/s13567-015-0216-x)

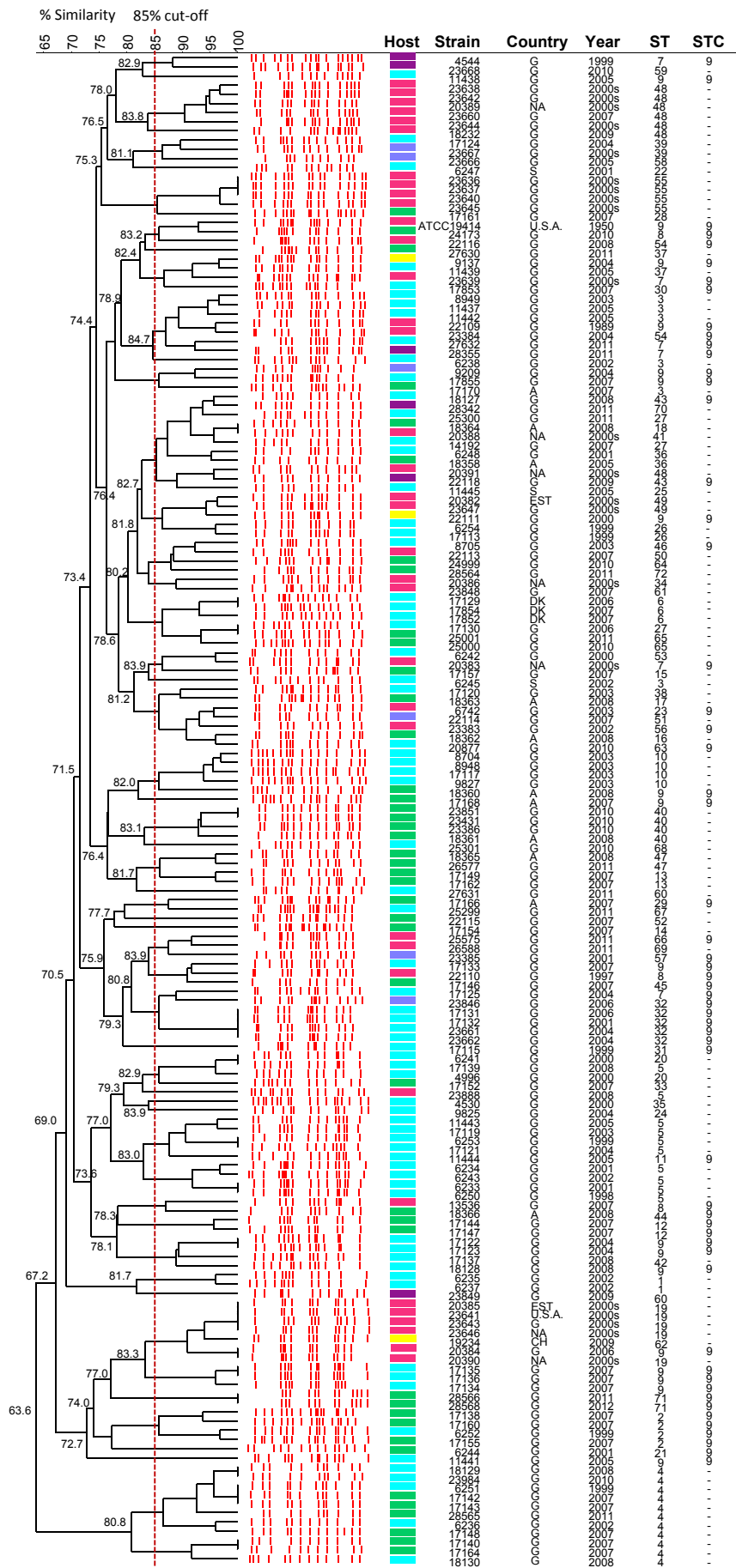

Supplement: Additional file 2: — Dendrogram for 165 field isolates from birds and mammals and the type strain of Erysipelothrix rhusiopathiae ATCC 19414 T , based on pulsed-field gel electrophoresis macrorestriction patterns generated by digestion with SmaI restriction endonuclease. This table provides PFGE banding patterns of 165 E. rhusiopathiae isolates with their sequence types (STs), ST complexes (if applicable), and epidemiological background (year, country, and host of isolation). Abbreviations: A = Austria, CH = Switzerland, DK = Denmark, EST = Republic of Estonia, G = Germany, NA = unknown, S = Sweden, USA = United States of America. [file 13567_2015_216_MOESM2_ESM.pdf]
